# Catalytic activity of novel thermoplastic/cellulose-Au nanocomposites prepared by cryomilling

**DOI:** 10.3906/kim-2005-53

**Published:** 2020-12-16

**Authors:** Joanna KWICZAK-YİĞİTBAŞI

**Affiliations:** 1 Department of Chemistry, Bilkent University, Ankara Turkey

**Keywords:** Mechanochemistry, cellulose-thermoplastic composites, gold nanoparticles, cryomilling

## Abstract

Due to environmental concerns, increasing attention has been focused on the application and preparation of biobased polymers and their blends. In this study, cellulose, the most spread biopolymer on Earth, was used in the preparation of novel cotton/polypropylene-Au and cotton/polyethylene-Au nanocomposites via a green mechanochemical approach. First, mechanoradicals were generated by ball milling of the cotton and thermoplastics under cryo conditions, and then, these radicals were used in the reduction of Au ions to Au nanoparticles (Au NPs). Nanocomposites were characterized by scanning electron microscopy (SEM), X-ray diffraction (XRD), and X-ray photoelectron spectroscopy (XPS). The application of mechanochemistry in obtaining the cotton/thermoplastic blends allowed homogenous and fine blending of the samples and in addition, excluded the usage of toxic solvents. Since Au NPs exhibit a wide range of applications, e.g., in catalysis, cotton/thermoplastic-Au nanocomposites were used to catalyze the reduction reaction of 4-nitrophenol to  4-aminophenol, followed by UV-Vis spectroscopy. Finally, the hydrophobicity of the nanocomposites was alternated by tuning the blend composition. In the prepared nanocomposites, cotton and thermoplastics acted as very good supporting matrices for the Au NPs and provided satisfactory access to the NPs.

## 1. Introduction

Cellulose, the most common polymer in nature, is built of *β* -D-glucopyranose units connected by *β* -1,4-glycosidic bonds [1,2]. This polysaccharide possesses desired properties, such as stiffness, low cost, strength, and high thermal stability [3]. Moreover, cellulose is obtained from sustainable and renewable sources, which in comparison to thermoplastics, has a positive impact on the environment. Thus, due to increasing problems connected with petroleum deficit and pollutions caused by synthetic polymers, cellulose stands out as a green alternative in obtaining sustainable materials. Indeed, the incorporation of cellulose as a nonabrasive and nontoxic matrix in its composites provides a low-density and low-weight final product [3,4]. Over recent years, special attention has been focused on the preparation of blends containing cellulose and thermoplastics, which can be beneficial, especially for the environment [5–10]. This has allowed the development of materials with improved properties, such as the introduction of cellulose fibers into synthetic polymers, which increased the strength and stiffness of the final product [6]. Moreover, incorporating thermoplastics into cellulose can change the surface properties; for example, hydrophobicity, which is crucial for applications in paper diagnostics [11–13]. Going one step further and developing cellulose/thermoplastic-metal nanocomposites provides hybrid materials exhibiting new advantageous properties coming from the metal nanoparticles [14,15]. Since nanoparticles have found wide applications in fields such as biology [16], medicine [16,17], and industry [18], cellulose-based metal nanocomposites also have begun to be used in paper diagnostics [13], catalysis [19], and as antimicrobial materials [20].

Preparation of cellulose/thermoplastic composites usually involves the use of twin screw extruder [5,6,8,9] or toxic solvents [7]. Thus, obtaining cellulose/thermoplastic-metal nanocomposites is usually an expensive and multistep process, consisting of the reduction of metal ions by reducing agents in a cellulose matrix, and then the incorporation of cellulose-metal nanoparticles into the thermoplastics [14,15]. Simpler and greener methods for the preparation of nanoparticles [21–23] and cellulose-metal, cellulose/thermoplastic-metal nanocomposites [12] have been proposedby mechanochemistry.The mechanochemical approach is usually an environmentally-friendly process that excludes the usage of hazardous and toxic reagents and solvents, and stands for green chemistry principles [24]. Moreover, techniques such as solid-state mechanical milling enable the preparation of highly mixed blends, often composed of immiscible polymers [25–29]. Enhancing the compatibilization and the degree of dispersion of the blends can be additionally achieved by increasing the milling time and conducting the milling process under cryogenic conditions [26–29]. Ball-milling can also be applied in the preparation of cellulose/thermoplastic composites, such as cellulose-maleated polypropylene and cellulose-maleated polyethylene, with improved mechanical properties in comparison to the same products formed by melt-mixing [30,31]. Thus, the cryomill arose as an alternative way of producing multifunctional polymeric materials, in which many problems faced in other blending strategies are avoided.

**Figure 1:**
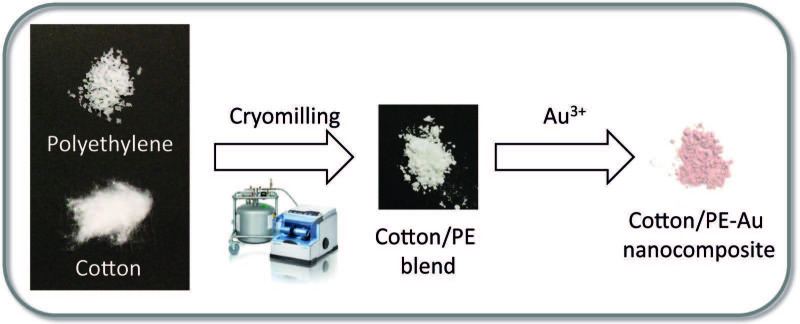
Preparation of the cotton/thermoplastic-Au nanocomposites. In the first stage, thermoplastic (PE or PP) and cotton were cryomilled for 30 min at 30 Hz at 77 K. Next, HAuCl4 solution in H2O (3 mL, 5.1 × 10–3 M) was added to the milled samples, resulting in the formation of Au NPs in the cotton/ thermoplastic matrices.

In the scope of this work, new cotton/polyethylene-, and cotton/polypropylene-Au nanocomposites were prepared by cryomilling (Figure 1). It has already been reported that, during the mechanochemical treatment of cellulose and other polymers, covalent bonds are broken, resulting in the formation of mechanoradicals [12,32–34]. Moreover, these radicals were found to be relevant in driving further reactions [12,34–36], or stabilizing chemical species on the polymer surface [37]. Similarly, herein, by the cryomilling of commercially available cotton and synthetic polymers, mechanoradicals were formed and used in the reduction of Au3+ to Au nanoparticles (Au NPs) (Figure 1). The resulting cotton/thermoplastic-Au nanocomposites were fully characterized by scanning electron microscopy (SEM), X-ray diffraction (XRD), and X-ray photoelectron spectroscopy (XPS). Recently, the preparation of robust and selective nanocatalysts has gained increasing attention [38,39]. Among them, cellulose-, and thermoplastic-based Au nanocomposites were reported as catalysts used in various organic transformations [19,40–42]. Nevertheless, the main concern in the preparation of these types of nanocatalysts is achieving the fine stabilization of Au NPs, and good deposition of small Au NPs, preventing them from aggregation. The main factors impacting the above issues are the structures and surface functional groups of the polymers supporting the nanoparticles [40–42]. Therefore, well-designed polymer matrices, which enable good access to Au NPs, area key parameter in conducting successful catalysis. In this work, the catalytic activity was also evaluated, and cotton/thermoplastic-Au nanocomposites were successfully involved in the catalytic transformation of 4-nitrophenol to 4‑aminophenol, showing the potential of cotton/PP and cotton/PE as nanoparticle matrices. Moreover, the surface properties of the prepared nanocomposites were studied via contact angle (CA) measurements. The incorporation of higher amounts of thermoplastics led to higher hydrophobicity of the blends.

## 2. Results and discussion

### 2.1. Synthesis and characterization of the cotton/thermoplastic-Au nanocomposites

**Figure 2:**
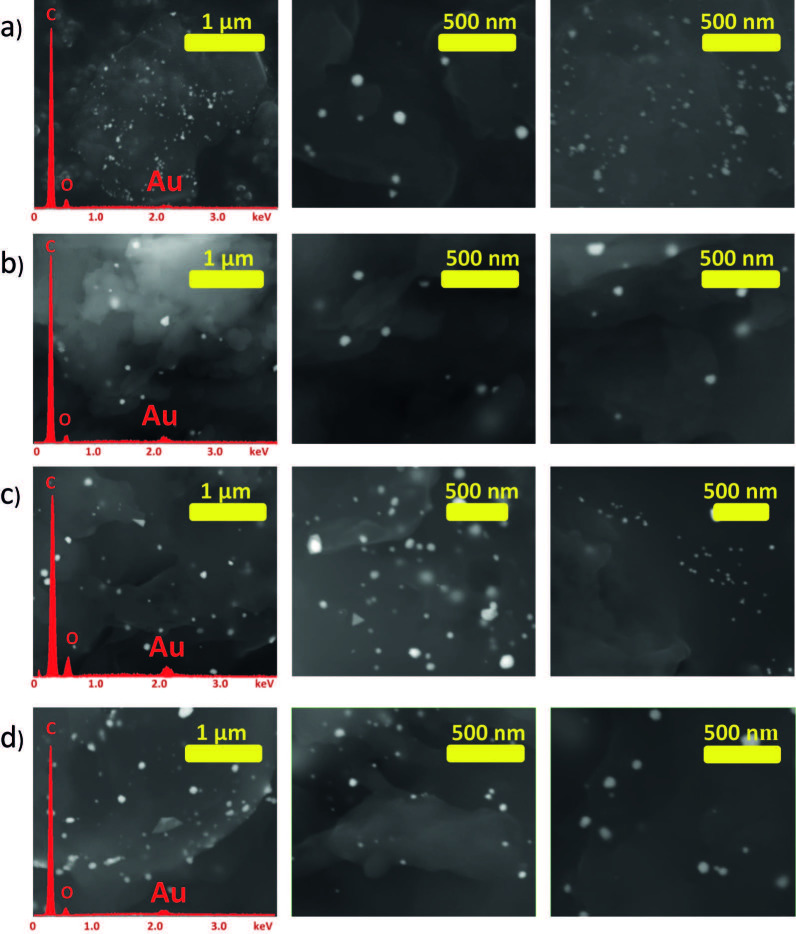
EDX spectra (shown in red) and SEM images of a) cotton/PE-Au 1, b) cotton/PE-Au 2, c) cotton/PP-Au 1, and d) cotton/PP-Au 2, prepared by grinding cotton and thermoplastic at 30 Hz and 77 K for 30 min, and then adding HAuCl4 solution in H2O (3 mL, 5.1 mM) (see Experimental section for more details).

Cotton/thermoplastic-Au nanocomposites were prepared by cryomilling cotton with corresponding polymer (cotton/thermoplastic ratio equal to 1:1 w/w (cotton-PP-Au 1, cotton-PE-Au 1) and 3:1 w/w (cotton-PP-Au 2, cotton-PE-Au 2)). It was previously reported that the highest amount of cellulose mechanoradicals is formed after 30 min of milling under cryo conditions (2.65 × 1018 radicals per gram of cotton) [43]. Moreover, the structural and morphological changes of cellulose, polyethylene, and polypropylene, caused by milling at low temperatures (reduction of the particle size and molecular weight, changes in crystallinity, etc.), were also previously studied in detail [43,44]. In a typical experiment, samples were milled at 30 Hz for 30 min. After milling, HAuCl4 solution in H2O (3 mL, 5.1 mM) was added onto ground cotton/thermoplastic samples and the mixtures were stored in the dark [at this point, it is important to mention that the control experiments were undertaken, nonmilled samples were left in the metal ion solutions, and no deposition of the metal NPs was observed, as verified by high-resolution XPS (HIRES XPS)]. In order to analyze the forming nanocomposites, small amounts of samples were taken after 1 and 2 weeks of waiting, and then the powders were washed to remove the remaining precursor solutions, and dried. It is worth mentioning that while storing the cotton/thermoplastic blends in the Au ion solutions, the color of the powders changed from white to purple, indicating the formation of Au NPs in the cotton/thermoplastic matrices. However, SEM images of cotton/thermoplastic-Au nanocomposites after 1 week of storing them in the Au ion precursor solutions, indicated very low concentration of Au NPs formed in the matrices. Therefore, in order to obtain higher deposition of the Au NPs, longer storage time was necessary. After 2 weeks of waiting, SEM images of the cotton/thermoplastic blends clearly showed the formation of nanoparticles as bright spots in the cotton/thermoplastic matrices (Figure 2). Nanoparticles were finely distributed in the blends and more importantly, they did not grow larger with longer waiting times. The lack of aggregation (even after weeks of storage) indicated that the nanoparticles were well-stabilized in the cotton/thermoplastic matrices. Indeed, it has been well-described that the porous structure of PE and PP has a stabilizing effect on nanoparticles [45], and as well, the porous cellulose matrix stabilizes nanoparticles as a result of strong secondary bonding between the NPs and the ether and hydroxyl groups present in cellulose [46].

**Figure 3:**
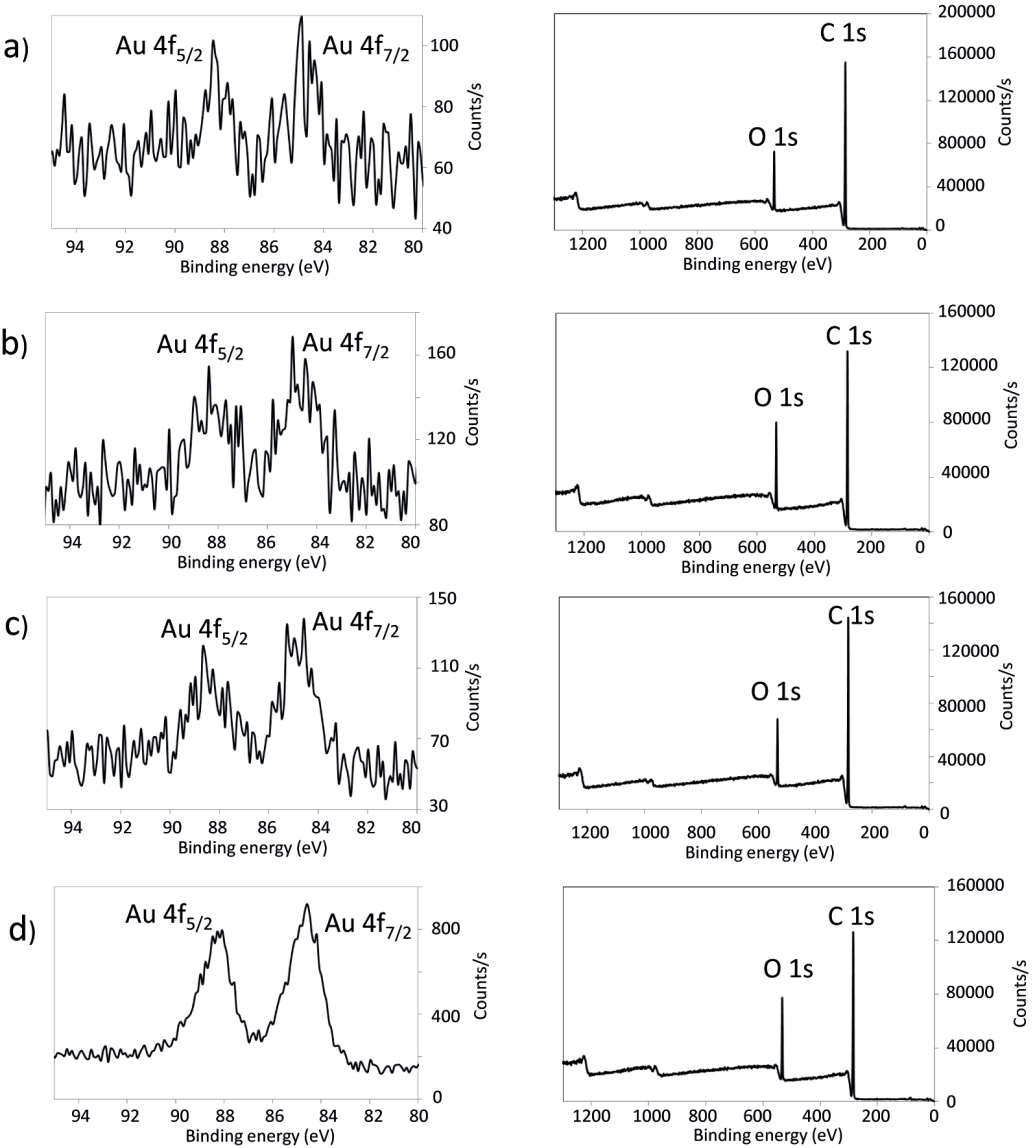
HIRES Au 4f XPS spectra (left column) and XPS survey (right column) of a) cotton/PE-Au 1, b) cotton/PE-Au 2, c) cotton/PP-Au 1, and d) cotton/PP-Au 2 nanocomposites, prepared by grinding cotton and thermoplastic at 30 Hz and 77 K for 30 min, and then adding HAuCl4 solution in H2O (3 mL, 5.1 mM) (see Experimental section for more details). These spectra confirmed the metallic nature of the Au NPs.

In order to confirm the deposition material in reduced metallic form, energy-dispersive X-ray spectroscopy (EDX) spectra, HIRES XPS, and XRD analyses were performed. Thus, the EDX spectra of the nanocomposites showed the presence of Au atoms (signal at 2.12 eV), and no signals that could be attributed to the corresponding counter ions in the metal ion precursors (Figure 2). The HIRES XPS spectra of the Au4f region are presented in Figure 3. For the cotton/PP-Au and cotton/PE-Au nanocomposites, 2 peaks arising from the spin orbit splitting of the Au 4f7/2 and 4f5/2 levels were observed. The binding energies of the Au 4f5/2 electrons appeared at approximately 87.88 eV, and the binding energies of the Au 4f7/2 electrons were at approximately 84.50 eV. These values matched the literature reports for Au in metallic state [47]. Additionally, no peaks coming from the Au3+ (86.9 eV and 90.6 eV) present in the Au ion precursors were detected [48,49].

XPS measurements were also used to estimate the atomic and weight percentages of the Au NPs present in the nanocomposites (Table 1). It turned out that the atomic percentages of Au in the cotton/PP-Au nanocomposites were slightly higher than those in the cotton/PE-Au nanocomposites, which may indicate that PP stabilized the Au NPs better than the PE matrix (Table 1).

**Table 1:** Atomic and weight percentages of Au in the cotton/thermoplastic-Au, and cotton‑Au nanocomposites, as determined by XPS (see Experimental for more details).

Nanocomposites	at.%	wt.%	Au content in 1.0 mg of nanocomposite (µg)
Cotton/PE-Au 1	0.020 ± 0.001	0.31	3.1
Cotton/PE-Au 2	0.034 ± 0.004	0.53	5.3
Cotton/PP-Au 1	0.059 ± 0.005	0.91	9.1
Cotton/PP-Au 2	0.055 ± 0.007	0.86	8.6
Cotton-Au	0.120 ± 0.025	1.69	16.9

**Figure 4:**
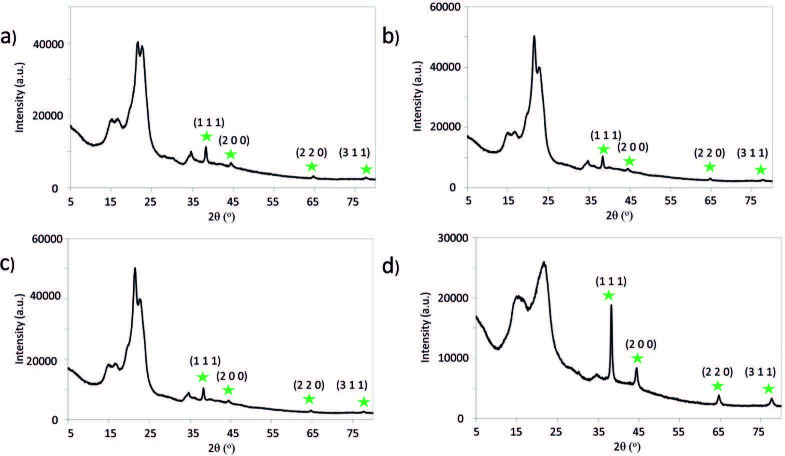
X-Ray diffractograms of a) cotton/PE-Au 1, b) cotton/PE-Au 2, c) cotton/PP-Au 1, and d) cotton/PP-Au 2 nanocomposites, prepared by grinding cotton and thermoplastic at 30 Hz and 77 K for 30 min, and then adding HAuCl4 solution in H2O (3 mL, 5.1 mM) (see Experimental section for more details). Diffractograms confirmed the metallic nature of the Au NPs, as shown by the presence of the signals at the star-labelled diffraction angles.

XRD analysis of the nanocomposites also supported the presence of the metallic state of Au (Figure 4). The diffraction lines of Au present in the cotton/thermoplastic-Au nanocomposites appeared at approximately 2θ: 38.2°, 44.3°, 64.5°, and 77.2°corresponding to the crystallographic planes of (1 1 1), (2 0 0), (2 2 0), and (3 1 1), respectively. These diffraction lines indicated the face-centered cubic structure of the metallic Au NPs [50].

**Figure 5:**
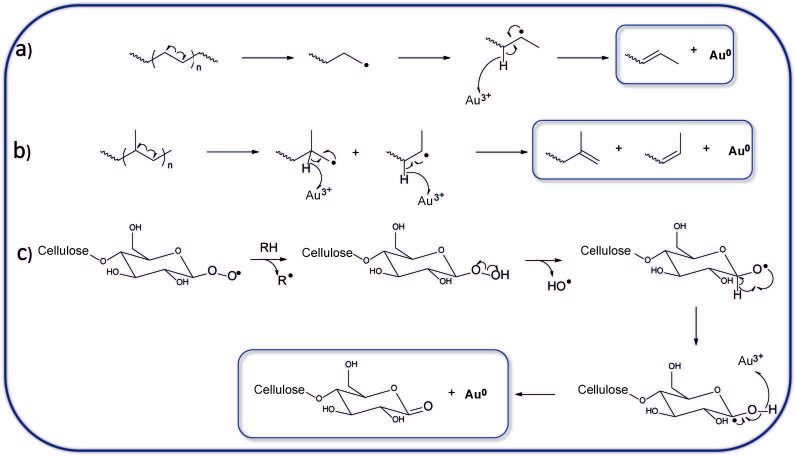
Mechanism of Au3+ reduction by mechanoradicals: a) primary polyethylene radicals formed upon cryomilling recombined to secondary radicals, which reduced the Au ions to Au NPs. b) Cryomilling of polypropylene caused the formation of radical pairs, which may reduce Au ions to their metallic form. c) First, cellulose peroxy radicals abstract H˙ from the CH2 and CH groups present in the cellulose. Next, unstable peroxides decomposed to alkoxy (CO˙), and hydroxyl (HO˙) radicals. Finally, alkoxy radicals converted to C-centered radicals, which reduced the Au ions to Au NPs [12].

The mechanism of metal ion reduction by cellulose radicals has already been reported [12]. Cellulose peroxy radicals undergo transformations leading to cellulose oxidation with the concurrent reduction of Au3+ to Au NPs (Figure 5) [12]. Milling of polyethylene causes the formation of primary radicals, which transform into more stable secondary radicals (Figure 5) [51]. These radicals may reduce Au3+ to Au NPs, but may also transform into peroxy radicals, which can also cause the reduction of Au ions, similar to cellulose radicals (Figure 5) [12,51].

**Figure 6:**
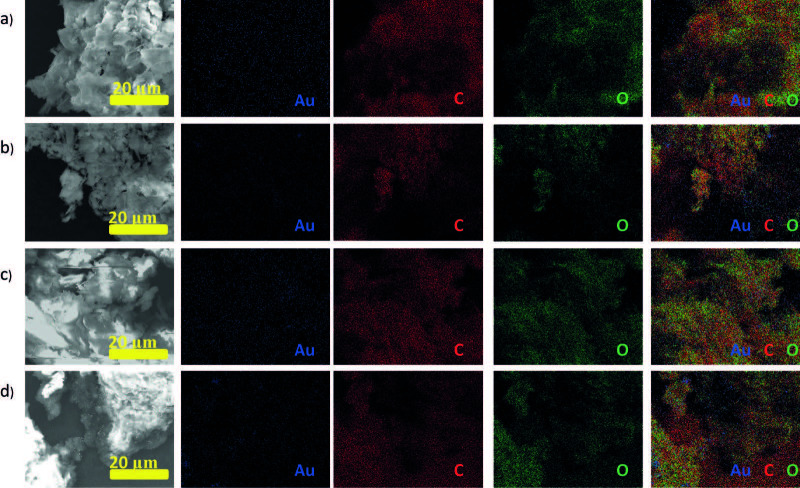
SEM-EDX elemental mapping of a) cotton/PE-Au 1, b) cotton/PE-Au 2, c) cotton/PP-Au 1, and d) cotton/PP-Au 2.

For future applications of nanocomposites, it is very important that cellulose and thermoplastic chains are well-mixed with each other and also, that Au NPs are equally distributed within the whole sample. Thus, to check whether fine distribution of the thermoplastics in the cellulose and Au NPs among the matrices takes place, elemental mapping by EDX-SEM analysis was performed (Figure 6). All of the maps confirmed the presence of C, O, and Au, and showed that the cotton and PP or PE mixed well with each other. Fine mixing of the components of the blends can be additionally supported by the XPS survey, in which peaks arising from both C 1s and O 1s were present, even after weeks of storage (Figure 3).

### 2.2. Wettability of the cotton/thermoplastic-Au nanocomposites

**Figure 7:**
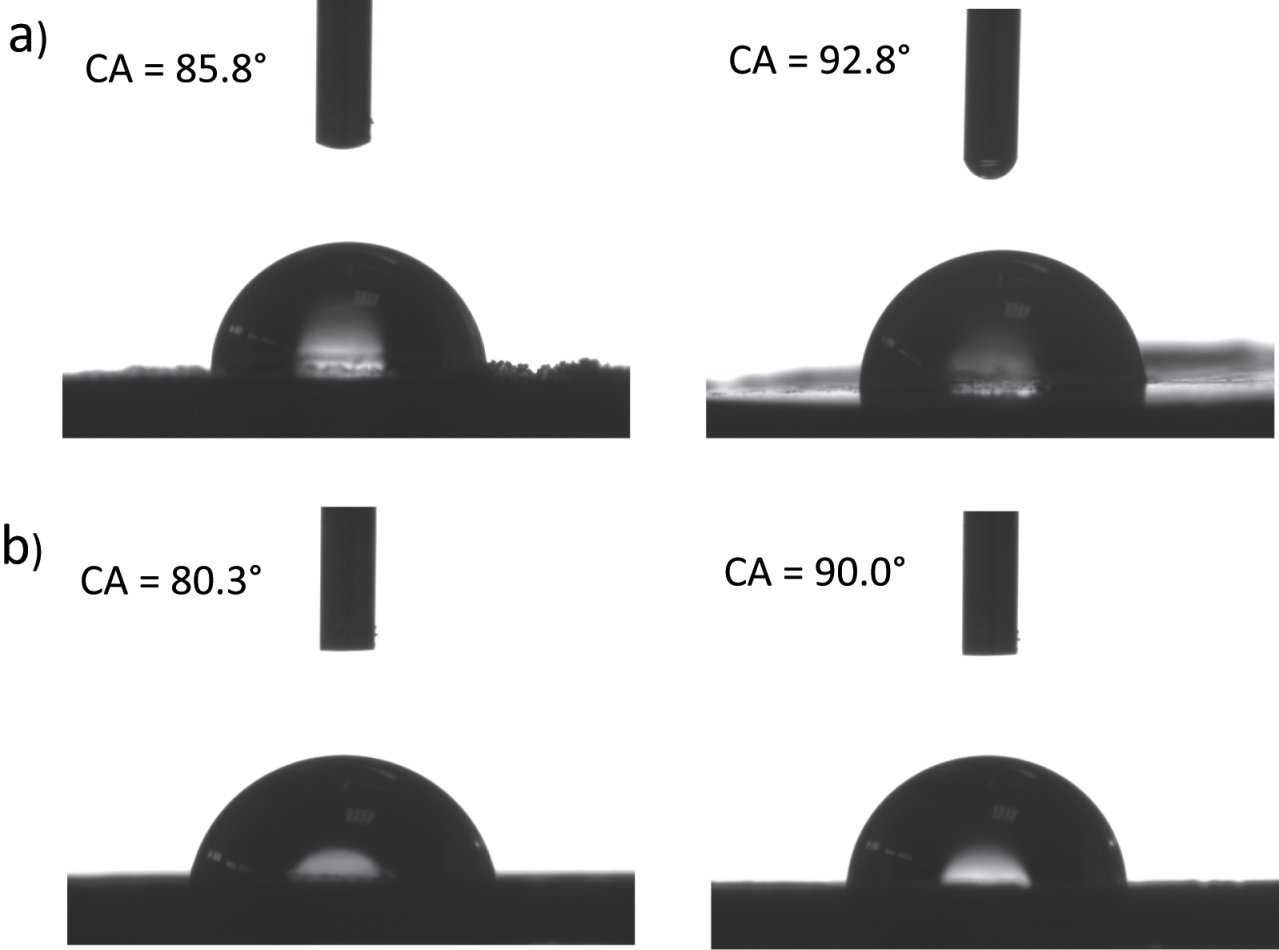
Water CA of a) cotton/PP-Au 2 (left), cotton/PP-Au 1 (right), and b) cotton/PE-Au 2 (left), cotton/PE-Au 1 (right).

It has already been shown that by the application of mechanochemistry, hydrophobicity of the blends containing cellulose and polystyrene can be regulated [12]. Tuning the surface properties of such nanocomposites can be crucial for their application in paper diagnostics [11,13]. Therefore, the same strategy was used herein, by blending the cotton with hydrophobic PP or PE with different weight ratios, it was possible to increase and tune the CAs. Thus, the CAs of cotton/PP-Au 2, and cotton/PE-Au 2 were equal to 85.8° ± 5.3° and 80.3° ± 4.6°, respectively (Figure 7). When the concentration of PE or PP increased to 50%,the CAs increased to 92.8° ± 2.8° for cotton/PP-Au 1 and 90.0° ± 4.2° for cotton/PE-Au 1 (Figure 7).

### 2.3. Catalytic activity of the cotton/thermoplastic-Au nanocomposites

The reduction of 4-nitrophenolate (4-NP) to 4-aminophenolate (4-AP) with NaBH4 serves as a model reaction in order to investigate the catalytic performance of metal nanoparticles [19]. This transformation was monitored by UV-Vis spectroscopy, where the decrease of the peak absorbance at 400 nm (corresponding to 4-NP) and the emergence of a new peak at 300 nm (corresponding to 4-AP) was followed [52–54]. The change of these characteristic peaks within the time was crucial to understand the rate of the reaction.

**Figure 8:**
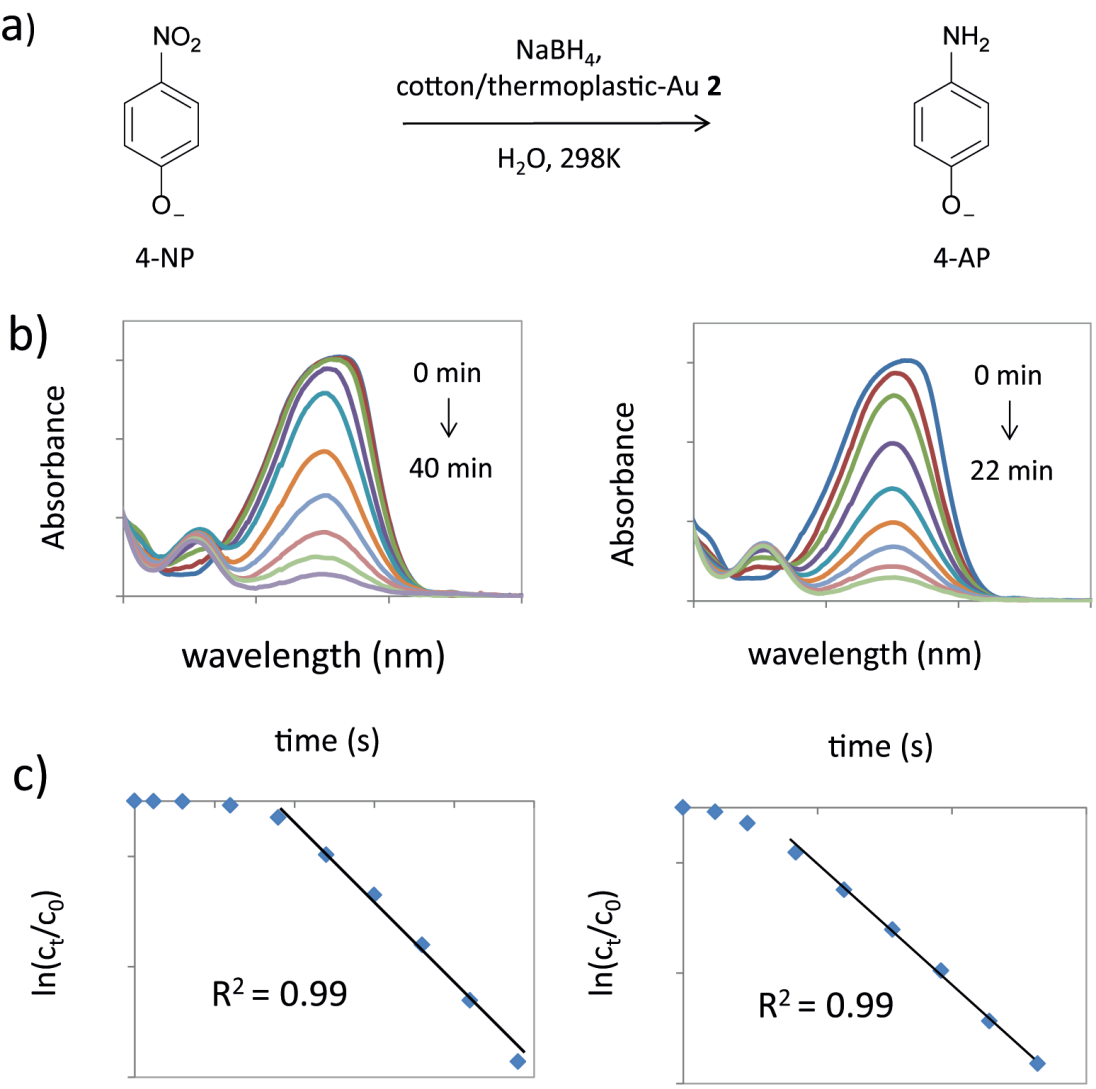
a) Transformation of 4-nitrophenolate to 4-aminophenolate with cotton/thermoplastic-Au 2 (1 mg of composite in powder form) used as a catalyst. b) UV-Vis absorption spectra during the catalytic cycle in the presence of cotton/PE-Au 2 (left) and cotton/PP-Au 2 (right). c) The reaction proceeds with pseudo-first order behavior, as indicated by the linear ln(Ct/C0) vs. time plots in the reaction catalyzed by cotton/PE-Au 2 (left) and cotton/PP-Au 2 (right).

In this study, the cotton/PP-Au 2 and cotton/PE-Au 2 powders were used as catalysts. Thus, in a typical experiment, NaBH4 (0.375 mL, 0.47 M in H2O) was added to a solution of 4-NP (3 mL, 0.30 mM in H2O). The mixture turned bright yellow, and a strong peak at 400 nm appeared, corresponding to the formation of 4-nitrophenolate ions (Figures 8a and 8b). After the introduction of the cotton/thermoplastic-Au nanocomposite powder (Au content in 1.0 mg of composite: 8.6 µg (cotton/PP-Au 2) and 5.3 µg (cotton/PE-Au 2)), the intensity of the absorption peak of 400 nm decreased, together with the appearance of a new peak at 300 nm caused by the conversion of 4-NP to 4-AP (Figure 8b). It is necessary to point out that the cotton/PP and cotton/PE powders did not have catalytic activity under the testing environment and the reduction of 4-NP in the presence of NaBH4 did not take place. Thus, the Au NPs supported on the cotton/thermoplastic matrix were needed to trigger the catalytic reaction [55].

Since the reduction was conducted under conditions where NaBH4 was used in great excess in comparison to the content of 4-NP, the reaction can be considered as independent of the NaBH4 concentration. Thus, it was postulated that the 4-NP transformation into 4-AP was following Langmuir-Hinshelwood mechanism, and it was a pseudo-first-order reaction [53,56].

Figure 8c provides the plots of ln(C/C0) against time (t) during the reduction of 4-NP, where C is the concentration of 4-NP at the designated time and C0 is the initial concentration of 4-NP at t = 0. A good linear correlation of ln(C/C0) vs. time indicated that the catalysis proceeded with pseudo-first-order behavior and also allowed the determination of the rate constant (k). Thus, the k values were equal to 0.0017 ± 0.0004 s–1 and 0.0013 ± 0.0002 s–1 for cotton/PP-Au 2 and cotton/PE-Au 2, respectively (Table 2, entries 3 and 4). Moreover, in these plots (Figure 8c), the induction period of the 4-NP reduction can be observed. The reason for that lies in the mechanism of the reaction, which was based on the adsorption of hydrides and 4-nitrophenol on the Au surface, followed by the reduction and desorption of the product [57]. The rate-determining step of this process was a B-H bond cleavage to give [Au]-H species, which were responsible for the reduction of the 4-nitrophenol. Since it took some time for [Au]-H to form, the reaction started after a delay, which caused the induction period. Additionally, the catalytic activity of the cotton/thermoplastic-Au nanocomposites was compared with other cellulose-based composites, which were prepared by conventional methods (Table 2). However, the constant rate of the 4-nitrophenol reduction catalyzed by metal nanoparticles depends on many factors, such as the type of polymeric support, and size and shape of the nanoparticles or stabilizing agents, which is why the comparison was only approximate [19,58]. Thus, the cotton/thermoplastic-Au nanocomposites displayed similar catalytic activity to the Au NPs supported on the cellulose nanocrystals (Table 2, entries 1 and 2). However, lower catalytic activity was observed in comparison to the Au NPs decorated on the cotton and microcrystalline cellulose (MCC) matrix (Table 2, entries 5 and 6), which might have been caused by 2 factors: a higher concentration of Au deposited on the cotton and MCC in comparison to the cellulose/thermoplastic nanocomposites, and lower wettability of the cellulose/thermoplastic blends than the hydrophilic MCC-, and cotton-supported Au NPs.

**Table 2:** Comparison of the cellulose-based nanocatalysts in the reduction of 4-nitrophenol.

Entry	Catalyst	Temperature (K)	Molar ratio			k (s–1)	Reference
			NaBH4	4-NP	Au		
1	CNC-PAMAM-Aua	298	5000	12.5	1	0.0020	Chen et al. [54]
2	AuNPs@CNsb	298	9720	30	1	0.0021	Wu et al. [59]
3	Cotton/PP-Au 2	298	6295	32	1	0.0017	Current study
4	Cotton/PE-Au 2	298	6518	33	1	0.0013	Current study
5	Cotton-Au	298	2039	10	1	0.0021	Current study
6	MCC-Auc	298	2200	11	1	0.0080	Kwiczak et al. [12]

aPolyamidoamine dendrimer grafted cellulose nanocrystals,bAu NPs on cellulose nanocrystals, cmicrocrystalline cellulose.

## 3. Experimental

### 3.1. Materials

Cotton was obtained from a local pharmacy. Hydrogen tetrachloroaurate trihydrate was purchased from abcr GmbH (Karlsruhe, Germany). Sodium borohydride, 4-nitrophenol, polypropylene (average MW = 280,000), and polyethylene (average MW = 550,000) were purchased from Sigma-Aldrich Corp. (St. Louis, MO, USA).

### 3.2. Instrumentation

#### 3.2.1.Cryomill

Milling under cryo conditions of the cellulose and thermoplastic samples was performed using a Retsch Cryomill with an integrated cooling system (Retsch GmbH, Haan, Germany).

#### 3.2.2. Scanning electron microscopy and energy dispersive X-ray analyses

The surface morphology of composites was imaged and analyzed with a Quanta 200F model SEM (Thermo Fisher Scientific Inc., Waltham, MA, USA) with an accelerating voltage of 15 kV.

#### 3.2.3. X-ray photoelectron spectroscopy

XPS spectra were recorded on an ESCALAB 250 Thermo Fisher Scientific K-Alpha X-ray photoelectron spectrometer. Photoemission was stimulated by a monochromatic Al K alpha radiation (1486.6 eV). Survey scans and high-resolution scans were collected using pass energies of 200 eV and 30 eV, respectively. Binding energies in the spectra were referenced to the C1s binding energy set at 284.8 eV.

#### 3.2.4. X-ray diffraction

XRD spectra were recorded on a X’Pert PRO Malvern Panalytical model X-ray diffractometer (Malvern Panalytical B.V., Almelo, Netherlands) with Cu K *α* radiation, 40 mA current, and 45 kV accelerating voltage.

#### 3.2.5. UV-Vis spectroscopy

The absorption spectra were recorded using a Cary 100 Bio UV-Visible spectrophotometer (Varian, Inc., Palo Alto, CA, USA).

#### 3.2.6. Contact angle

CA measurements were recorded on the Contact Angle System OCA from Data Physics Corp. (San Jose, CA, USA).

### 3.3. Methods

#### 3.3.1. Preparation of the cotton/thermoplastic-Au nanocomposites

First, 300 mg of cotton and thermoplastic (polypropylene or polyethylene) were cryomilled at 77 K for 30 min at 30 Hz in the presence of 6 zirconia balls (with 10.06 mm diameter) in a zirconia sample chamber. Next, 3 mL, 5.1 × 10–3 M of HAuCl4 solution in H2O was added to the chamber and the mixture was mixed at 5 Hz for 30 s. After that, the mixture was diluted to 6 mL with H2O, placed in a polypropylene tube, and stored in dark for 14 days. Finally, the solid was washed with H2O and dried.

#### 3.3.2. Atomic and weight percentage calculation of Au NPs in the nanocomposites

Atomic percentages (at.%) of Au in the nanocomposites presented in Table 1 were calculated based on the area of the peak evaluated from the XPS survey for each nanocomposite. The following sensitivity factors were used for the calculations: 2.881 (O1s), 1.000 (C1s), and 20.735 (Au4f). The atomic percentage was an average of at least 5 independent XPS measurements. The weight percentage (wt.%) was calculated by the following equation:

wt.%x=(at.%Au).(at.wt.Au)(at.%Au).(at.wt.Aux)+(at.%C).(at.wt.C)+(at.%O).(at.wt.O).100%

where at.% Au is the atomic percentage of Au in the nanocomposite, at.% C is the atomic percentage of C in the nanocomposite, at.% O is the atomic percentage of O in the nanocomposite, at.wt. Au is the atomic weight of Au, at.wt. C is the atomic weight of C, and at.% wt. O is the atomic weight of O.

#### 3.3.3. Reduction of 4-nitrophenol

To a solution of 4-NP in H2O (0.30 mM, 3 mL), a solution of NaBH4 in H2O (0.47 M, 0.375 mL) was added. Next, 1.0 mg of cotton/thermoplastic-Au nanocomposite [total metal content: 8.6 µg (cotton/PP 2), 5.3 µg (cotton/PE 2)] was added. The reaction was conducted in a quartz UV cuvette at room temperature and monitored using a UV-Vis spectrophotometer. Standard deviation was calculated based on at least 3 independent measurements.

#### 3.3.4. Contact angle measurements

First, 70 mg of cotton/thermoplastic-Au nanocomposite powders were pressed into pellets. Next, the CAs of 3 µL water drops on the pellet surfaces were measured 10 times for each pellet at room temperature.

## 4. Conclusion

In this work, the preparation of novel cotton/PP-Au and cotton/PE-Au nanocomposites was demonstrated. In the first stage, cellulose and thermoplastic mechanoradicals were formed by cryomilling, which were used afterwards in the reduction of the Au ions into Au NPs. This method excluded the usage of toxic solvents, and in the preparation of the nanocomposites, only water was used. The Au NPs were well-stabilized in the cotton/thermoplastic matrices, and did not aggregate, even after weeks of storage. Moreover, the incorporation of thermoplastics into the cotton matrix with 2 different weight ratios changed the wetting abilities of the samples, increasing the concentration of thermoplastics from 25% to 50%, and increased the CAs of the nanocomposites. In addition, the cotton/PP-Au and cotton-PE-Au nanocomposites were successfully used as catalysts in the reduction of 4-nitrophenol to 4-aminophenol. The transformations occurred with satisfactory reaction constant rates.
